# Evaluation of Sperm Retrieval Efficiency and Extender Impact in Cryopreserved Canine Epididymal Semen

**DOI:** 10.3390/vetsci12090840

**Published:** 2025-08-31

**Authors:** Elisabeth Bernklau, Axel Wehrend, Abbas Farshad

**Affiliations:** Veterinary Clinic for Reproductive Medicine and Neonatology, Justus-Liebig-University of Giessen, 35392 Giessen, Germany

**Keywords:** sperm, epididymis, cryopreservation, incubation, diluent

## Abstract

(1) Background: Cryopreservation of epididymal spermatozoa in dogs is challenging due to their lower cryotolerance compared to ejaculated spermatozoa. Given the limited sperm volume obtained per individual, efficient recovery and preservation techniques are essential. (2) Methods: This study assessed sperm collection and cryopreservation methods from the cauda epididymis of ten dogs undergoing routine elective castration. After dissection and mincing, the cauda epididymidis tissue was incubated in 0.9% saline at 38 °C for either 10- or 30-min. Samples were analyzed for concentration and motility using AndroVision^®^ software (CASA; AndroVision™; Minitüb GmbH) (Tiefenbach, Germany). Additional evaluations included histological examination, hypoosmotic swelling test, live/dead staining, and morphological assessments. Three extenders, custom-made Tris-Fructose-Citrate (Tris), custom-made Uppsala, and commercial Optixcell^®^, were used for cryopreservation and compared for post-thaw sperm quality. (3) Results: No significant differences were found between the 10- and 30-min incubation groups regarding sperm motility, viability, or histological integrity. The total sperm counts were 292 × 10^6^ ± 175 × 10^6^ for the 10 min group and 233 × 10^6^ ± 162 × 10^6^ for the 30 min group (*p* = 0.56). Histological sections revealed no significant difference in residual intraluminal spermatozoa between groups, indicating that 10 min of incubation is sufficient for effective sperm migration. Post-thaw sperm motility was significantly higher with Uppsala (17.2 ± 12.2%) and Optixcell^®^ (11.7 ± 6.5%) compared to Tris (4.7 ± 4.8%). Morphological abnormalities were lowest in samples preserved with Optixcell^®^ (37.5 ± 10.1%, *p* = 0.005). (4) Conclusion: A 10 min incubation period is adequate for efficient recovery of epididymal sperm in dogs. Among the tested extenders, Uppsala and Optixcell^®^ demonstrated superior cryoprotective effects, resulting in better post-thaw motility and reduced morphological abnormalities compared to Tris.

## Simple Summary

Cryopreservation of epididymal sperm in dogs is challenging due to their lower freezing tolerance compared to ejaculated sperm, making efficient recovery important. This study evaluated sperm collection using 10 or 30 min incubation of dissected cauda epididymis tissue in saline. Both times yielded similar sperm counts, motility, and viability, suggesting 10 min is sufficient. Three extenders, Tris, Uppsala and Optixcell^®^, were tested for freezing efficiency. Uppsala and Optixcell^®^ resulted in better post-thaw motility and fewer abnormalities, making them more effective than Tris for preserving sperm quality.

## 1. Introduction

Refining and preserving epididymal spermatozoa are essential for advancing artificial insemination in canines, particularly when natural mating is not an option [1]. This process supports the evaluation of sperm vitality, morphology, and motility [2], thereby improving reproductive outcomes [3,4,5,6]. Notably, harvesting and cryopreserving epididymal spermatozoa often represent the final opportunity to preserve male breeding potential in mammals. Furthermore, preparation techniques such as filtration and centrifugation play a critical role in determining sperm quality [7].

Within this context, spermatozoa collection and incubation techniques following castration were explored [8,9], while sperm separation through centrifugation and assessment of membrane integrity were undertaken [10,11,12]. In one study, epididymal spermatozoa from 23 euthanized dogs were obtained and evaluated for longevity during storage at both room temperature (22–24 °C) and 4 °C, using fluid aspiration with a 25 G needle into TALP medium [13], whereas another approach employed a 26 G needle with a vacuum syringe containing Ham’s F-10 medium enriched with supplementary components [5]. In parallel, epididymal spermatozoa were retrieved from a 9-year-old Boxer post-euthanasia by flushing the ductus deferens with saline [14], and a comparable technique, using a Gent^®^ semen extender, was applied to collect spermatozoa from an American Staffordshire Terrier post-castration [15]. Building on these approaches, the cauda epididymidis and ductus deferens were flushed with a Tris–fructose–citrate solution supplemented with Equex STM Paste [16,17], while the efficacy of Biladyl^®^ and Andromed^®^ extenders, in combination with prostatic fluid administered during and after flushing, was also evaluated [18]. Expanding on this methodology, an incubation step followed by flushing with either Tris or ACP-106c extender was implemented [19], which was later refined by incorporating prostatic fluid during flushing to further enhance sperm quality [20]. Furthermore, the influence of different anesthetic protocols on sperm retrieval was examined using a Tris–glucose–citrate buffer for flushing the ductus deferens and cauda epididymidis [21]. Collectively, these studies reflect the progressive refinement of standardized and precise collection methods aimed at ensuring optimal spermatozoa preservation.

While many researchers avoid additional processing prior to analysis, others employ filtration or centrifugation to enhance sample purity. For example, sperm suspensions have been filtered to remove tissue debris, centrifuged using a Percoll^®^ gradient (manufactured by Cytiva, Marlborough, MA, USA), and subsequently incubated with concentration adjustments [8,9]. Comparable filtration-based strategies have also been reported [22,23]. In another approach, the sperm suspension was centrifuged, resuspended in Brackett and Oliphant solution, and subjected to a Percoll^®^ gradient [12]. Additional techniques include purification through nylon filtration [24] and centrifugation of extended spermatozoa samples [19].

With respect to spermatozoa storage and cryopreservation, the fertilization capacity of frozen–thawed epididymal spermatozoa has been evaluated using either 0.9% NaCl or Ringer’s solution [25]. A subsequent investigation compared a cattle-based extender with the conventional Tris–citrate–glucose–egg yolk–7% glycerol formulation, following storage of testes in saline at 5 °C for 24 h prior to collection [26]. To minimize desiccation, sterile saline and anatomical segmentation of the epididymis have been applied, together with cryopreservation in extenders such as egg yolk–Tris–fructose–citrate and prostatic fluid [20,23,27,28,29,30]. Similarly, Tris–fructose–citrate combined with Equex STM Paste has been employed [16,17], while the comparative performance of Biladyl^®^ and Andromed^®^ extenders—with or without prostatic fluid—has also been assessed for their influence on post-thaw sperm quality [31,32]. Collectively, these findings emphasize that accurate collection, careful processing, and optimized cryopreservation are critical for maintaining viability and fertilization capacity. In particular, evidence indicates that motile spermatozoa can still be recovered after several days of cooled storage [8], underscoring the value of meticulous handling in preserving spermatozoa integrity for successful artificial insemination This study aims to evaluate the factors influencing the efficiency of cryopreserving canine epididymal sperm, with the central hypothesis that both the incubation time during sperm retrieval and the choice of cryoprotective extender significantly affect post-thaw sperm quality and quantity; therefore, the objectives are to assess how varying incubation times impact sperm yield and quality prior to cryopreservation, to compare the effectiveness of three different extenders for freezing the sperm, and ultimately to establish a standardized protocol for the collection and cryopreservation of canine epididymal sperm.

## 2. Materials and Methods

### 2.1. Ethical Approval and Reagents

The study involving animal experimentation was approved by the local ethics committee via the Animal Welfare Office of Justus Liebig University Giessen, in accordance with Institutional Review Board protocol kTV 8-2017, dated 30 May 2017. All chemicals and reagents used in the study were obtained from the following German suppliers: Sigma-Aldrich Chemie GmbH (Steinheim, Germany), Carl Roth GmbH (Karlsruhe, Germany), Merck KGaA (Darmstadt, Germany), and Merck Schuchardt OHG (Hohenbrunn, Germany).

### 2.2. Epididymal Sperm Collection and Experimental Design

**Figure 1:**
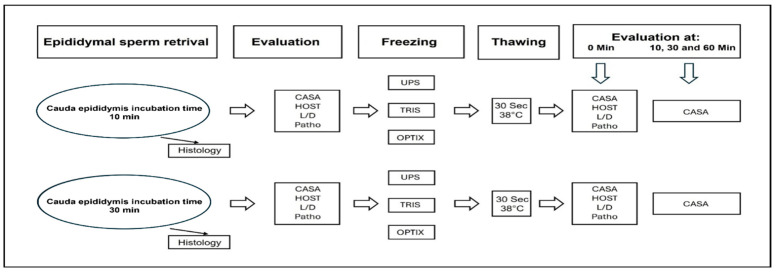
Overview of the processes for sperm collection, preparation, and analysis, including CASA (Computer-Assisted Sperm Analysis), HOST (Hypoosmotic Swelling Test), L/D (Live–Dead Assay), Patho (Pathomorphological Evaluation), UPS (Uppsala), and Optix (Optixcell^®^).

Epididymides were collected from ten clinically healthy male dogs of various breeds, including Labrador Retriever, Jack Russell Terrier, German Shepherd, Papillon, Beagle, Chihuahua, Australian Shepherd, and mixed breeds. The dogs ranged in age from 1.3 to 7.0 years (mean: 2.86 years) and weighed between 4.0 and 40.5 kg (mean: 20.79 kg). Neutering was performed under general anesthesia, induced with Midazolam (1 mg/kg) and Levomethadone (0.5 mg/kg), and maintained with Isoflurane via endotracheal intubation. Both epididymides, along with the testes and tunica vaginalis, were surgically removed through a pre-scrotal incision. Immediately after excision, tissues were placed in sterile 0.9% sodium chloride solution to maintain hydration and prevent desiccation [25]. The epididymides were then separated from the testes and carefully cleaned of connective tissue using a scalpel. The cauda epididymidis was minced in 5–10 mL of sterile 0.9% sodium chloride solution (volume adjusted to tissue size) in a glass container. One cauda from each dog was incubated for 10 min (protocol 1) and the other for 30 min (protocol 2) at 38 °C. Following incubation, the suspensions were filtered through a 0.7 mm metal sieve to remove residual tissue debris. As shown in Figure 1, the sperm suspension was evaluated and prepared for cryopreservation using Uppsala, TRIS, and Optixcell^®^ freezing extenders. Subsequently, sperm samples were analyzed using multiple techniques, including histological assessment of cellular structure and integrity, measurements of motility and concentration, hypoosmotic swelling (HOS) tests, live/dead viability staining, and pathomorphological evaluation.

### 2.3. Preparation of Dilutions and Cryopreservation of Semen Samples

#### 2.3.1. Uppsala Diluent

The Uppsala diluent, prepared in-house, is a two-part extender system used for sperm sample preparation, designed to achieve a final concentration of 1.0 × 10^8^ sperm per mL with a dilution ratio of 2:1. It consists of two formulations, UPS 1 and UPS 2, each with distinct chemical compositions and physical properties. UPS 1 is prepared by dissolving 3.025 g of Tris, 1.7 g of citric acid, and 1.25 g of fructose in distilled water up to 77 mL. To this base, 3 mL of glycerol, 20 mL of egg yolk, and 0.8 g of Equex paste are added. The resulting solution has a pH of 6.72 and an osmotic pressure of 865 mOsm. UPS 2 follows a similar formulation but uses 72 mL of distilled water and increases the glycerol content to 7 mL, producing a slightly higher pH of 6.74 and an osmolarity of 1495 mOsm. For sperm processing, the sperm pellet was first resuspended at room temperature using half of the total volume of UPS 1. This was achieved by gradually adding the diluent drop by drop along the edge of a glass container while gently swirling, using a plastic pipette or syringe. Following this, the sample underwent an equilibration phase for one hour at 4 °C in the refrigerator. During this time, the remaining volume of UPS 2, pre-cooled to 4 °C, was added in the same manner.

#### 2.3.2. Tris Diluent

The Tris diluent is used in sperm sample preparation and consists of two components of cryodiluent (Cryodiluent 1 and Cryodiluent 2) and a sperm diluent. The preparation begins by mixing the sperm diluent with half of the total volume of the diluent at room temperature. This mixture is then transferred into a beaker, and the rim of the glass is gently warmed to prevent the liquid from sticking to the surface. Afterward, the mixture is cooled to 4 °C, and Cryo-diluent 1 is added. The solution is then cooled again to 4 °C in the refrigerator. Following this, the remaining volume of Cryodiluent 2 is added, and the entire mixture is cooled once more to 4 °C and stored at that temperature until use.

#### 2.3.3. The Optixcell^®^ Diluent

The Optixcell^®^ diluent (IMV Technologies, Saint Ouen Sur Iton, France) is prepared according to the manufacturer’s instructions. Initially, the diluent is warmed to 34 °C and then mixed with twice the amount of distilled water, also heated to 34 °C. Any unused portions of the diluent are deep-frozen and thawed shortly before use. For sperm sample preparation, the sperm pellet is resuspended by gradually adding the full volume of Optixcell^®^ diluent drop by drop using a pipette. The mixture is gently stirred and then equilibrated for three hours at 34 °C.

#### 2.3.4. Cryopreservation of Semen Samples

Spermatozoa from one epididymis were divided into three equal portions in graduated centrifuge tubes and centrifuged at 700× *g* for 6 min (Type 1703, Hettich, Germany). After removing the supernatant, pellets were resuspended in UPS, Tris, or Optixcell^®^ extender. Pellets with fewer than 1.0 × 10^8^ sperm were diluted to 1 mL, each straw contains 2.5 × 10^7^ sperm, and those with higher counts to 2 mL. At 20–25 °C, half the diluent volume was added dropwise at the tube edge with gentle swirling, then mixed using a plastic pipette (Eppendorf, Hamburg, Germany). Samples equilibrate at 4 °C for 1 h in a refrigerator. The remaining precooled diluent was then added similarly. Suspensions were loaded into precooled 0.25 mL straws (Minitüb, Tiefenbach, Germany), sealed with balls (Minitüb), and held for 10 min in liquid nitrogen vapor at 4–5 cm distance from the surface of liquid nitrogen before submersion in liquid nitrogen (–196 °C). After at least 24 h, straws were thawed in a 37 °C water bath for 30 s (Tun 1052, Gesellschaft für Labortechnik mbH, Burgwedel, Germany).

### 2.4. Examination of Epididymal Sperm Before Freezing Process

**Figure 2:**
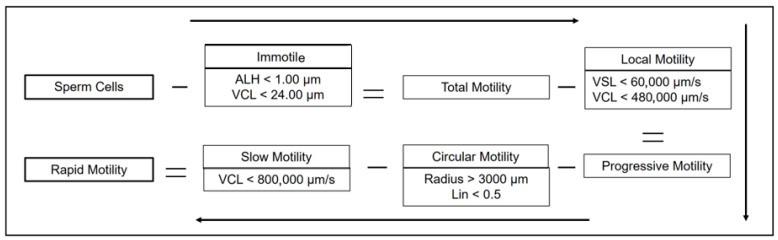
Sperm motility classification flowchart using minus signs to indicate reductions and equal signs to show resulting motility groups. ALH: amplitude of lateral head displacement; VCL: curvilinear velocity; VSL: straight-line velocity.

Sperm motility was assessed, as illustrated in Figure 1, using Computer-Assisted Sperm Analysis (CASA) with AndroVision™ (Minitüb GmbH, Tiefenbach, Germany), both prior to extension and following thawing, in accordance with the manufacturer’s canine-specific protocols. To ensure consistency across samples, 3000 spermatozoa or 15 microscopic fields per sample were analyzed using Leja chambers (Leja Products B.V., Nieuw-Vennep, The Netherlands) featuring a depth of 20 µm and a maximum volume of 3 µL, which were maintained at 37 °C after a 30 s settling period. Subsequently, Figure 2 outlines the evaluated parameters, which encompassed total, progressive, rapid, circular, slow, and local motility, as well as immotile sperm and sperm concentration (×10^6^/mL), collectively offering a detailed profile of sperm quality. In addition, motility subtypes were derived from sperm trajectory data, with specific metrics including amplitude of lateral head displacement (ALH), indicating side-to-side head movement (µm); curvilinear velocity (VCL), representing average speed along a curved path (µm/s); and straight-line velocity (VSL), reflecting velocity along a linear path (µm/s). Complementary parameters such as linearity (VSL/VCL ratio) and radius further elucidated the efficiency and orientation of sperm motion. Taken together, these data points enabled a comprehensive characterization of sperm motility and its relevance to fertilization potential.

To assess sperm membrane integrity, 10 µL of sperm solution was added to 100 µL of hypoosmotic swelling (HOS) test solution, prepared by dissolving 0.375 g sodium citrate and 1.351 g fructose in 100 mL of distilled water. The mixture was incubated in an Eppendorf tube at 37 °C for 30 min. After incubation, a coverslip was placed on the sample and examined under a microscope (H500, Hund GmbH, Wetzlar, Germany) at 400× magnification. Spermatozoa with intact membranes exhibited tail curling in the hypoosmotic solution, while those showing no tail response or fully coiled tails were considered defective [33].

Viable spermatozoa were evaluated using eosin staining. The stain was prepared by dissolving 2 g eosin-G and 3 g sodium citrate in 100 mL of distilled water. For the assay, 10 µL of eosin solution was mixed with a larger drop of sperm on a pre-warmed slide and left for 20 s. A smear was then made on a second warmed slide (IDL GmbH & Co KG, Nidderau, Germany), air-dried, and 200 spermatozoa were examined at 400× magnification. Eosin staining also allowed detailed pathomorphological assessment. Using oil immersion microscopy at 1000× magnification (H500, Hund GmbH), 200 spermatozoa were analyzed for primary abnormalities (head, acrosome, neck and tail defects), as well as secondary and tertiary abnormalities such as loose heads and tail coiling. In addition, the presence of cytoplasmic droplets was recorded, with only proximal droplets considered, since distal droplets are not classified as abnormalities.

### 2.5. Analysis of Epididymal Sperm After Freezing and Thawing

Post-thaw sperm motility was assessed at intervals of 10, 30, and 60 min following thawing at 37 °C, utilizing the same Computer-Assisted Sperm Analysis (CASA) protocol employed for pre-freeze evaluations. Additionally, hypo-osmotic swelling test (HOST), eosin–nigrosin viability staining, and pathomorphological assessments were conducted in accordance with the procedures previously applied to epididymal sperm prior to cryopreservation.

For histology, samples were placed in embedding cassettes and fixed in 10% formalin for ≥7 d. After fixation, tissue samples were incubated in sodium phosphate buffer and stored in 70% ethanol for 24 h. Dehydration was performed using increasing alcohol concentrations (80%, 96%, 100%) for 15 min each, followed by xylene immersion. Paraffin embedding was completed using an embedding machine, and blocks were cooled at 4 °C for 24 h. Sections (3 µm) were cut using a microtome, floated in a 38 °C water bath, and mounted on APES-coated slides. Slides were dried at 38 °C for 24 h in an incubator and stored at room temperature in the dark until it was stained.

**Figure 3:**
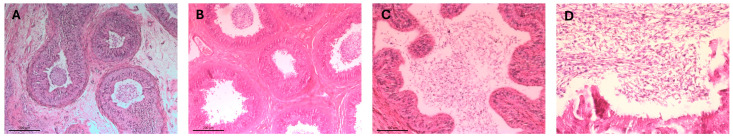
Histological sections of epididymal duct in the tail region of the epididymis, (**A**–**D**) present H&E-stained histological sections of the canine epididymal tail; (**A**) shows intact tubule with lumens densely filled with spermatozoa. (**B**) displays variable sperm presence, ranging from absent to moderate across different sections and (**C**) reveals minimal sperm within the lumen accompanied by signs of epithelial disruption. (**D**) shows tubules with more consistently visible spermatozoa, indicating improved sperm retention.

**Figure 4:**
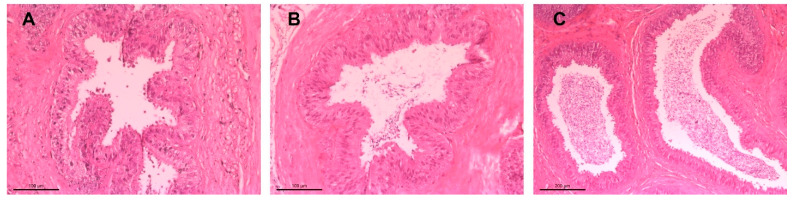
Histological sections of the epididymal duct in the tail region of the epididymis, stained with hematoxylin-eosin (HE): (**A**) absence of sperm, (**B**) few spermatozoa at 20× magnification, and (**C**) abundant spermatozoa at 10× magnification.

An overview staining was performed with hematoxylin and eosin (HE). Sections were first deparaffinized in xylene (20 min), rehydrated through graded alcohol into distilled water, then stained with hematoxylin (5 min), followed by rinsing under tap water (15 min). Eosin staining was performed for 5 min, and excess stain was removed via rinsing in tap water, 80%, and 96% alcohol. Staining quality was checked at 100× magnification and adjusted if needed by dipping in ethanol. The final dehydration step was carried out using alcohol and xylene, followed by mounting the sections with Entellan^®^ New and cover slips. After solidification at room temperature, the preparations were examined under a light microscope (Figure 3). Each epididymal tail section was categorized into three groups based on sperm content: no sperm, few sperm, and many sperm (Figure 4).

### 2.6. Statistical Analysis

Statistical analyses were performed using Microsoft^®^ Office Excel^®^ 2013 and the BMDP statistical package (Release 8.1, 2000). BMDP/1D was used for descriptive statistics, BMDP/3D for dependent sample t-tests, BMDP/2V for variance analyses, and BMDP/60 for scatterplot generation. The analysis compared two collection protocols and three cryopreservation extenders for canine epididymal spermatozoa. Descriptive statistics included arithmetic means, standard deviations (SD), standard errors, and minimum–maximum values. Logarithmic transformations were applied to non-normally distributed data. A paired t-test was used to compare protocols 1 and 2, whereas analysis of variance evaluated differences among cryopreservation systems, considering protocol, diluent, and time. Statistical significance was set at *p* < 0.05.

## 3. Results

Table 1 summarizes the evaluated parameters of native epididymal spermatozoa collected after 10 min (protocol 1) and 30 min (protocol 2) incubation. Each parameter is reported as an arithmetic mean ± SD, along with its minimum and maximum observed values. Parameters include total motility, progressive motility, rapid progressive motility, circular motion, slow motility, local motility, immotile spermatozoa, sperm concentration, total sperm count, percentage of spermatozoa with intact membranes (HOS test), percentage of viable spermatozoa (via live–dead staining), and pathologically altered forms. Although both protocols showed similar trends, protocol 1 consistently produced higher mean values for total motility (70.0% vs. 65.8%), progressive motility (64.2% vs. 59.3%), and live sperm percentage (68.4% vs. 64.8%). In contrast, protocol 2 showed marginally higher percentages of immotile spermatozoa (34.2% vs. 30.0%) and rapid progressive spermatozoa (26.1% vs. 25.9%), alongside a reduction in pathological forms (24.3% vs. 27.5%).

Moreover, sperm concentration and total sperm count were higher in protocol 1 (40.9 × 10^6^/mL and 291.6 × 10^6^, respectively) than in protocol 2 (32.47 × 10^6^/mL and 233.3 × 10^6^). These differences suggest that incubation time may influence motility, viability, and total sperm yield. Finally, histological examination of the epididymal tail revealed that 34.2% of cross-sections had no spermatozoa, 24.7% had a limited amount, and 41.1% contained a substantial quantity, as illustrated in Figure 3A–C. In addition, analysis of epididymal spermatozoa quality at different incubation intervals demonstrated that cryopreserved sperm samples incubated for 10 and 30 min exhibited superior post-thaw motility parameters when diluted with Uppsala and Optixcell^®^ extenders compared to those of Tris as described in Table 2, Table 3 and Table 4. The corresponding data, including arithmetic means and standard deviations, were documented immediately after thawing.

Regarding the incubation protocols, a three-factor variance analysis revealed no significant influence on motility parameters measured by CASA (Total Motility: *p* = 0.44; Progressive Motility: *p* = 0.24; Rapid Progressive Motility: *p* = 0.83; Circular Motility: *p* = 0.60; Slow Motility: *p* = 0.11; Stationary Motility: *p* = 0.62), Table 4. However, there was a consistent trend toward lower values at nearly every time point for protocol 2 compared to that of protocol 1. Additionally, total sperm count was higher in protocol 1, with a borderline non-significant difference (*p* = 0.054). In contrast, motility parameters were significantly higher for spermatozoa diluted with Uppsala (total motility: *p* < 0.001; progressive motility: *p* < 0.001; rapid progressive motility: *p* < 0.001; circular motility: *p* < 0.001; slow motility: *p* < 0.001; local motility: *p* < 0.001) and Optixcell^®^ (total motility: *p* < 0.001; progressive motility: *p* = 0.008; rapid progressive motility: *p* = 0.006; circular motility: *p* = 0.009; slow motility: *p* = 0.01; local motility: *p* < 0.001) compared to that of Tris. The differences between Uppsala and Optixcell^®^ were not significant, except for circular motility (*p* = 0.028). Futhermore, the live–dead staining results were significantly better for Uppsala (*p* < 0.001) and Optixcell^®^ (*p* = 0.01) compared to those of Tris. Similarly, HOS test results were higher for Uppsala (*p* < 0.001) and Optixcell^®^ (*p* = 0.003). The average proportion of morphologically abnormal spermatozoa was significantly lower in the Optixcell^®^ group compared to that of Uppsala (*p* = 0.008), whereas differences relative to Tris were not significant (*p* = 0.057). These findings demonstrate that Uppsala and Optixcell^®^ extenders significantly enhance motility and membrane integrity relative to Tris, with Optixcell^®^ producing a lower incidence of morphological abnormalities than Uppsala.

## 4. Discussion

For various reasons, collecting epididymal spermatozoa from domestic dogs or wild canids for in vivo fertilization or cryopreservation may be necessary. Several studies have examined the collection and preservation of canine epididymal spermatozoa, revealing lower tolerance to cryopreservation compared to ejaculated spermatozoa, particularly in terms of motility parameters [20,23,29,34]. Nevertheless, successful pregnancies and births have been achieved using fresh epididymal spermatozoa via intravaginal insemination [24] and cryopreserved epididymal spermatozoa via intrauterine insemination [14,23]. The primary aim of this study was to refine and optimize the process of epididymal sperm collection to increase the efficiency of subsequent processing and application. In addition, the study included a comparative evaluation of three cryopreservation diluents: two self-developed Tris egg yolk diluents (Tris and Uppsala diluents) and one commercially available option (Optixcell^®^). Although the Uppsala diluent effectively preserves canine sperm in both research and clinical settings, this study introduces a novel approach to assessing its performance compared to other Tris-based diluents and the commercially available Optixcell^®^ diluent specifically designed for canine sperm.

Furthermore, this study aimed to investigate whether the incubation period, defined as the time spermatozoa were allowed to swim out of the epididymis into the surrounding medium, affects both the quality parameters and the total number of recovered epididymal sperm, with the overarching objective of optimizing sperm yield for subsequent processing and practical application. In this context, the discussion on motility parameters and total epididymal sperm count reveals considerable variations influenced by the methods and conditions employed. Studies have demonstrated significant differences in motility and recovery rates, which are affected by factors such as collection techniques and the composition of media used [8,10,14,21,22,23,24,35,36,37,38]. Achieving high motility rates has been linked to specific breeds and controlled experimental conditions [39,40], highlighting the critical need for standardized methodologies to ensure consistent and reliable results. The primary aim of this study was to refine and optimize the epididymal spermatozoa collection process to improve subsequent processing and application. Additionally, the study examined how incubation time, defined as the duration during which spermatozoa migrate from the epididymis into the surrounding medium, affects sperm quality and yield.

A comparative evaluation of three extenders was also performed: two self-formulated Tris-egg yolk diluents (Tris and Uppsala) and one commercial extender (Optixcell^®^). Although the Uppsala extender showed efficacy in preserving canine spermatozoa in both research and clinical settings, this study presents a novel comparison with another Tris-based extender and Optixcell^®^, which had not been previously tested for canine epididymal spermatozoa. Furthermore, the study assessed whether the spermatozoa’s incubation time impacts post-thaw quality and recovery efficiency. The overarching goal was to maximize the epididymal spermatozoa yield for further reproductive use. In this context, discussion of motility parameters and total sperm counts highlights considerable variation associated with methodologies and incubation conditions. Differences in motility and recovery rates are influenced by factors such as collection technique and media composition [8,10,14,21,22,23,24,35,36,37,38].

Sperm motility outcomes are influenced by multiple factors, including the method of sperm retrieval and the composition of the collection medium. Techniques such as cutting, incubation, squeezing, or aspirating the cauda epididymis have been shown to affect motility parameters [20]. Additionally, the use of media enriched with buffers and sugars plays a critical role in preserving sperm function during collection and processing [3,4,5,10]. High motility rates have also been linked to breed-specific traits and consistent experimental conditions, highlighting the importance of standardized protocols in reproductive studies [39,40]. The buffer and sugar composition of the collection medium also significantly impacts spermatozoa performance [3,4,5,10]. Epididymal spermatozoa quality varies substantially, with reported motility values differing widely across studies [30]. Prior work suggests that incubation time has minimal effect on sperm parameters when comparing both epididymides from the same dog.

Dog size and age are known to influence sperm output, with total spermatozoa recovered correlating positively with body weight [39,40] and age-related changes affecting sperm yield [35]. In our study, the considerable variation in age and body weight among individuals likely contributed to differences in sperm recovery. Additionally, 25.9% of spermatozoa exhibited pathological alterations, including 20.5% with cytoplasmic droplets—an indicator of incomplete sperm maturation. It should be noted that histological evaluation was performed after tissue mincing at both incubation time points; however, the extent of tissue fragmentation and fixation may have influenced the visibility and quantification of sperm within the epididymal lumen. These factors, combined with the lack of comparison to alternative sperm retrieval techniques such as flushing or aspiration, represent limitations that may have impacted the consistency and interpretability of the histological findings. Literature reports altered spermatozoa ranging from 7.8% [20] to 73% [26], and immature forms from 1.6% [20] to 56% [23]. Functional assays yielded plasma membrane integrity of 68% (HOS test) and viability of 66.4% (eosin G), aligning with previously reported viability ranges of 32.5% to 94% [8,10,14,21,22,23,24,35,36,37,38]. Reported HOS test results for plasma membrane integrity ranged from 75.1% to 84.2% [10,41]. In this regard, there is a report [36] that presents lower membrane integrity in epididymal spermatozoa (69.2%) compared to ejaculated spermatozoa (90.2%), attributing this to the absence of catalase, a critical antioxidant [42,43]. Histological analysis revealed no significant differences in spermatozoa presence in the ductus epididymidis, aligning with native spermatozoa counts. However, sperm loss during fixation and embedding may have influenced these observations.

Analysis of cryopreserved spermatozoa motility parameters showed significant variation based on the diluent used. Total motility averaged 17% for Uppsala, 12% for Optixcell^®^, and 5% for Tris. Although low, these results are consistent with prior data ranging from 5% [14] to 69.3% [26]. Some studies preselect high-motility spermatozoa to improve results [16,26]. The higher sensitivity of epididymal spermatozoa to freezing is attributed to residual cytoplasmic droplets, morphological immaturity, and the lack of prostatic secretions [8,16,27,31,34,38]. Studies suggest that incorporating prostatic fluid enhances motility, viability, and fertility [28], although it may impair chromatin integrity [37]. In contrast, the saline-based medium used in this study lacked energy substrates or buffers, potentially contributing to reduced motility due to energy depletion. Post-thaw morphological analysis showed 43.5% altered forms, within mid-range literature values [29,34]. Among diluents, Optixcell^®^ yielded the lowest proportion of abnormalities (37%), compared to that of Tris (44.9%) and Uppsala (48.8%), suggesting superior membrane protection.

The average plasma membrane integrity via HOS was 14.0%, with significant differences among diluents (*p* < 0.002), highest for Uppsala and lowest for Tris. Live–dead staining yielded similar findings (14.1%, *p* < 0.001). CASA post-thaw showed motility improvements at 10 min with Protocol 2 across diluents, whereas Protocol 1 showed increases only with Tris. This suggests energy depletion during prolonged incubation may be offset post-thaw by the presence of exogenous energy sources. After the initial increase, motility declined in all groups. Incubation time had no significant effect on fresh sperm motility; however, the 10 min protocol consistently outperformed the 30 min one in post-thaw vitality and motility, likely due to reduced metabolic exhaustion. Likewise, pre-processing incubation time (10 vs. 30 min at 37 °C) had no significant effect on cryopreserved motility outcomes. Still, total motility favored Protocol 1 for all diluents: Uppsala (18.9% vs. 15.3%), Tris (5.1% vs. 4.3%), and Optixcell^®^ (14.0% vs. 9.3%). Sperm counts were 291.6 ± 179.0 × 10^6^ for Protocol 1 and 233.3 ± 162.0 × 10^6^ for Protocol 2, with no significant difference observed (*p* = 0.054). Extending the incubation time did not increase sperm yield and may have contributed to sperm clumping or adherence to epididymal tissue remnants.

## 5. Conclusions

This study underscores the importance of refining incubation protocols and selecting appropriate cryopreservation extenders to enhance the quality of canine epididymal spermatozoa for reproductive use. Shorter incubation times were associated with improved motility and viability, likely due to reduced energy depletion during pre-processing. Among the tested extenders, Optixcell^®^ demonstrated the most protective effect during cryopreservation, with lower proportions of morphologically altered spermatozoa compared to Tris and Uppsala. Although the Uppsala extender showed high plasma membrane integrity, its overall performance alongside Tris highlighted opportunities for refinement. These findings affirm that incubation duration, collection methodology, and extender composition are critical variables in optimizing epididymal spermatozoa recovery and cryopreservation outcomes. This study contributes valuable insight into improving sperm preservation techniques, supporting both clinical and research applications in canine reproductive medicine.

## Figures and Tables

**Table 1:** Comparison of sperm parameters at 10 and 30 min incubation. Values are presented as mean ± standard deviation (SD), along with the observed minimum and maximum ranges.

Parameter	10 min (Mean ± SD)	30 min (Mean ± SD)	10 min/30 min (Min–Max)
Total Motility (%)	70.0 ± 19.8	65.8 ± 18.0 *	35.0–93.3/29.9–86.7
Progressive Motility (%)	64.2 ± 20.9	60.0 ± 18.0	29.3–89.7/25.7–79.9
Rapid Progressive (%)	26.1 ± 11.9	26.0 ± 10.2 **	8.0–4.7/9.6–42.2
Circular Motion (%)	22 ± 12	18.0 ± 7.0	1.0–1.8/5.0–4.1
Slow Motility (%)	15.6 ± 8.2	15.0 ± 8.3	3.0–3.3/4.0–33.0
Local Motility (%)	6.8 ± 5.5	6.0 ± 1.9 *	6.0–25.0/3.9–10.9
Immotile (%)	30.0 ± 1.9	34.2 ± 18.0	6.7–65.0/13.3–70.1
Concentration (10⁶/mL)	292 ± 175	233 ± 162 **	6.0–26.5/7.0–73.0
HOS Test (%)	71.2 ± 17.2	66.0 ± 18.0	40.0–92.0/38.03–92.0
Live-Dead Staining (%)	71.0 ± 17.0	66.2 ± 18.0	40.0–92/38.0–92.0

TM: Total motility, PM: Progressive motility, RM: Rapid progressive motility, CM: circular motility, SM: Slow motility, LM: Localized motility; Asterisks denote statistically significant differences between the 10 min and 30 min time points, with * indicating *p* < 0.05 and ** indicating *p* < 0.01.

**Table 2:** Epididymal spermatozoa characteristics follow 10 min (protocol 1) and 30 min (protocol 2) incubation in different extenders. Data presented as Mean ± SD.

	Incubation Time					
	10 Min.			30 Min.		
Parameters	Uppsala	Optixcell	Tris	Uppsala	Optixcell	Tris
Host Test (%)	18.2 ± 12.4	15.6 ± 9.3	10.3 ± 8.6	17.8 ± 7.6	13.4 ± 5.8	41.5 ± 8.6
Live/Dead (%)	20.9 ± 14.1	15.5 ± 9.3	9.4 ± 12.0	20.1 ± 10.5	10.3 ± 5.3 *	8.3 ± 6.9
Pathomorphology (%)	53.6 ± 9.7	39.4 ± 11.5	48.3 ± 9.4	43.8 ± 9.9 *	41.5 ± 8.6	7.8 ± 9.9

Each row corresponds to a specific motility parameter, and values are shown across time points for all three diluents. Asterisks (*) indicate statistically significant differences between the 60 min and baseline (0 min) measurements within the same diluent group; Significance indicators: * = *p* < 0.05.

**Table 3:** The mean values ± standard deviation (SD) of various cryopreserved sperm motility parameters measured at 0, 10, 30 and 60 min after 10 min incubation time under three different diluent conditions: Uppsala-diluent, Tris-diluent, and Optixcell^®^-diluent.

	Uppsala-Diluent				Tris-Diluent				Optixcell^®^-Diluent			
	0 Min.	10 Min.	30 Min.	60 Min.	0 Min.	10 Min.	30 Min.	60 Min.	0 Min.	10 Min.	30 Min.	60 Min.
TM (%)	19.0 ± 4.6	17.9 ± 5.2	12.8 ± 13.3	8.6 ± 1.5 *	5.1 ± 4.8	5.6 ± 5.7	4.5 ± 4.5	3.5 ± 2.8	14.1 ± 8.8	13.6 ± 8.5	8.3 ± 4.2	5.3 ± 1.6 **
PM (%)	13.2 ± 4.1	11.8 ± 4.5	8.2 ± 9.4	5.4 ± 2.4 *	3.0 ± 3.5	3.3 ± 3.7	2.4 ± 2.7	1.7 ± 1.5	8.9 ± 6.7	8.1 ± 6.0	3.9 ± 2.2	2.1 ± 0.9 *
RM (%)	3.6 ± 3.2	3.6 ± 5.2	3.2 ± 1.4	2.3 ± 1.9	0.9 ± 1.3	0.7 ± 1.1	0.5 ± 0.6	0.3 ± 0.3	2.4 ± 12.1	2.2 ± 1.8	0.8 ± 0.5	0.4 ± 0.2
CM (%)	3.5 ± 1.3	3.5 ± 1.3	2.3 ± 1.2	1.4 ± 1.1	0.2 ± 0.3	0.2 ± 0.2	0.1 ± 0.2	0.1 ± 0.2	2.0 ± 2.0	1.2 ± 1.3	0.3 ± 0.3	0.1 ± 0.1
SM (%)	7.6 ± 2.5	7.2 ± 2.7	4.1 ± 1.4	2.3 ± 1.2 *	2.0 ± 1.9	2.4 ± 2.4	1.8 ± 1.9	1.3 ± 1.0	4.6 ± 3.0	4.7 ± 3.2	2.8 ± 1.5	1.3 ± 0.4 *
LM (%)	5.7 ± 3.5	5.2 ± 3.5	4.4 ± 1.4	3.1 ± 1.1	2.1 ± 1.3	2.3 ± 2.1	2.1 ± 1.9	1.8 ± 1.4	5.2 ± 2.3	5.5 ± 2.6	4.4 ± 2.0	3.2 ± 0.9

Each row corresponds to a specific motility parameter, and values are shown across time points for all three diluents. Asterisks (*) indicate statistically significant differences between the 60 min and baseline (0 min) measurements within the same diluent group; Significance indicators: * = *p* < 0.05. ** = *p* < 0.01.

**Table 4:** The mean values ± standard deviation (SD) of various cryopreserved sperm motility parameters measured at 0, 10, 30, and 60 min after 30 min incubation time under three different diluent conditions: Uppsala-diluent, Tris-diluent, and Optixcell^®^-diluent.

	Uppsala-Diluent				Tris-Diluent				Optixcell^®^-Diluent			
	0 Min.	10 Min.	30 Min.	60 Min.	0 Min.	10 Min.	30 Min.	60 Min.	0 Min.	10 Min.	30 Min.	60 Min.
TM (%)	15.3 ± 9.7	15.7 ± 8.2	8.8 ± 5.1	5.4 ± 4.2 *	4.3 ± 4.8	5.1 ± 4.6	3.4 ± 2.0	2.9 ± 1.3	9.21 ± 4.3	10.4 ± 6.7	7.4 ± 3.6	5.5 ± 2.4 *
PM (%)	10.3 ± 7.5	10.6 ± 6.4	5.5 ± 4.0	3.0 ± 2.3 *	2.6 ± 3.8	3.2 ± 4.1	1.7 ± 1.5	1.4 ± 1.0	4.8 ± 3.0	5.5 ± 4.7	3.1 ± 2.3	2.2 ± 1.3 *
RM (%)	2.7 ± 2.5	3.0 ± 1.9	1.3 ± 1.0	0.7 ± 0.7	0.6 ± 1.2	0.9 ± 1.5	0.4 ± 0.6	0.4 ± 0.5	1.1 ± 0.8	1.4 ± 1.3	0.7 ± 0.6	0.4 ± 0.4
CM (%)	1.7 ± 1.9	1.5 ± 1.4	0.5 ± 0.4	0.4 ± 0.6	0.3 ± 0.6	0.3 ± 0.5	0.2 ± 0.2	0.2 ± 0.3	1.0 ± 0.9	0.7 ± 0.8	0.3 ± 0.4	0.1 ± 0.1 *
SM (%)	5.9 ± 3.4	6.2 ± 3.7	3.6 ± 2.8	1.9 ± 1.6 *	1.8 ± 2.0	2.1 ± 2.1	1.2 ± 0.7	0.9 ± 0.3	2.7 ± 1.4	3.4 ± 2.7	2.1 ± 1.4	1.7 ± 0.9 *
LM (%)	5.0 ± 2.7	5.0 ± 2.1	3.3 ± 1.4	2.5 ± 1.5 *	1.7 ± 1.0	1.9 ± 0.8	1.7 ± 0.6	1.5 ± 0.4	4.5 ± 1.4	4.8 ± 2.2	4.2 ± 1.6	3.3 ± 1.3

Each row corresponds to a specific motility parameter, and values are shown across time points for all three diluents. Asterisks (*) indicate statistically significant differences between the 60 min and baseline (0 min) measurements within the same diluent group. * = *p* < 0.05.

## Data Availability

The authors retain ownership of the data produced in this study, which can be obtained by contacting the corresponding author.
